# Regionalization of the Mortality Risk from Cardiomyopathy and Respiratory Diseases Based on the Maximum Entropy Model

**DOI:** 10.1155/2024/6103589

**Published:** 2024-08-20

**Authors:** Raymundo Ordoñez-Sierra, Gabriela Domínguez-Cortinas, Iván Yassmany Hernández-Paniagua, José Luis Expósito-Castillo, Miguel A. Gómez-Albores, María Guadalupe Rodríguez-Reyes, Brisa Violeta Carrasco-Gallegos, Luis Ricardo Manzano-Solís

**Affiliations:** ^1^ Instituto Interamericano de Tecnología y Ciencias Del Agua Universidad Autónoma Del Estado de México, Carretera Toluca-Atlacomulco, Km 14.5, Toluca, Estado de México, Mexico; ^2^ Facultad de Geografía Universidad Autónoma Del Estado de México Cerro de Coatepec Sin Número Ciudad Universitaria, Toluca 50110, Estado de México, Mexico; ^3^ Universidad Autónoma de San Luis Potosí, Venustiano Carranza, San Luis Potosí 78210, Mexico; ^4^ Instituto de Ciencias de la Atmósfera y Cambio Climático Circuito de la Investigación Científica S/N Universidad Nacional Autónoma de México, C.U., Coyoacán, Ciudad de México 04510, Mexico

## Abstract

This research presents a time-series study in one of the most polluted regions in Mexico, the southern part of the Mezquital Valley. Three mortality causes related to areas highly contaminated by industrial activities were considered to carry out this model, namely, ischemic cardiomyopathy, mesothelioma, and pneumoconiosis. The pollutant exposure factors used in the maximum entropy modeling were distance to rivers, distance to industries, particulate matter less than 2.5 microns (PM < 2.5 *µ*m), and the digital elevation model (DEM). A model that expresses the presence of the disease by areas of exposure to pollutants was also obtained. In addition, the odds ratio was calculated to evaluate the level of association of ischemic cardiomyopathy (OR = 3.37 and 95% CI: 3.05–3.6) and mesothelioma (OR = 4.79 and 95% CI: 3.5–6.08) by areas of exposure. In the case of pneumoconiosis, only cases in the very high exposure category were recorded, so it was not comparable with the remaining areas. It is important to mention that particulate matter in the municipalities of the Mezquital Valley presented values above 20 *μ*g/m^3^ and that in accordance with the provisions of the Norma Oficial Mexicana de Salud Ambiental or NOM (translated as Mexican Official Standard for Environmental Health) and the Agency for Toxic Substances and the Disease Registry (ATSDR), high concentrations of particulate matter can have a severe impact on the development of some diseases. In the studied area, ischemic cardiomyopathy and mesothelioma were attributed to pollution in 70.3% and 79.1%, respectively; therefore, pollution mitigation could prevent the occurrence of these two diseases.

## 1. Introduction

Heavy industry and expansion of megacities involve extensive infrastructure that generate high concentrations of air and water pollution [1], such as wastewater treatment and waste incineration plants, transportation networks, and the combination of the heavy industry sector and anthropogenic activities, thus increasing the risk of developing acute and severe diseases [2] in the populations residing near these areas [3].

The most polluting industries generate emissions of toxic gases, and the same time discharge waste into bodies of water, that generally contain volatile organic compounds. These activities cause carcinogenic diseases due to the presence of sulfur, nickel, cadmium, and magnesium, among others [4]. It is estimated that particulate matter suspended in the environment causes 7 million deaths each year [5], whilst 1.8 billion people consume contaminated water, causing cardiovascular and respiratory diseases, malnutrition, and an increase in premature deaths. Water contamination is associated with gastrointestinal diseases, typhoid fever, and poliomyelitis [6, 7].

In Mexico, industries related to mining, petrochemicals, and metallurgy, among others, are established in the central part of the country; of these, power plants and refineries are characterized by the burning of fossil fuels [8]. The predominant use of fuel oil during this process releases high concentrations of sulfur into the air [9], causing significant risks to both the environment and human health.

Various groups such as scientists, citizens, journalists, photographers, academics, and activists exposed part of the health crisis that Mexico is facing by touring the central part of the country. This revealed that some regions are under a health and environmental emergency due to their environmental deterioration and the development of diseases. Among the regions with the greatest impact is the one located within the Mezquital Valley [10] where intense industrial activity with potential health risks is concentrated [11]. Mezquital Valley covers the municipalities of Tula de Allende, Atotonilco de Tula, Atitalaquia, Tepeji del Río de Ocampo, and Tequixquiac in the State of Hidalgo, and Huehuetoca and Apaxco in the State of Mexico. This region has experienced a variety of environmental problems, such as the discharge of wastewater from Mexico City, open-pit mining processes, and the establishment of a refinery and thermoelectric plant in municipalities within the State of Hidalgo [12]. Large concentrations of heavy metals such as lead, cadmium, and nickel [13] among other types of waste have been found in the wastewater and soil (in the first 20 centimeters) throughout the Hidalgo region. These waters are used in rainfed agriculture that uses the flood irrigation method [14], an activity that represents 59% of the total production for the entire region [15].

According to the National Statistical Directory of Economic Units (DENUE in Spanish), 8 out of 36 cement manufacturing plants that exist nationwide are located in the Mezquital Valley. In this region, there is also a refinery and a thermoelectric plant. Of the latter type, the PEMEX (a portmanteau of Petroleos Mexicanos which translates to Mexican Petroleum in English) plant emits 173,428 tons/year of sulfur dioxide (SO_2_) and 16,937 tons/year of nitrogen oxide (NO_2_), whilst the Comisión Federal de Electricidad or CFE (translated as Federal Electricity Commission in English) plant is the fifth largest in the country, emitting around 150,700 tons/year of SO_2_ and 16,361 tons/year of NO_2_ [1]. In addition to environmental pollution, other factors become apparent, such as material shortages and insufficient health infrastructure, as well as economic and cultural factors [16, 17]. The aim of the study was to generate a mortality risk regionalization for both respiratory and cardiac diseases based on a maximum entropy model for the southern area of the Mezquital Valley.

### 1.1. Spatial Exposure Model Based on Maximum Entropy

The potential geographic distribution modeling techniques based on maximum entropy have had a variety of applications with the intention to improve the knowledge of both the dynamics of and the presence of diseases [18–20]. The evaluation of the probability of occurrence of these models is done using the performance measurement area under the curve known as the receiver operating characteristic (AUC-ROC). This type of evaluation compares errors of omission and commission [21].

The application of these models has demonstrated to be a useful tool to understand the diseases' spatial distribution and, when combined with epidemiological indicators of frequency and association, as a way to quantify the risk in the population [22, 23]. These models also support the identification of those sites that, based on their pollution conditions, may give rise to areas with high probabilities for the development of diseases.

It is important to understand the behavior of diseases related to environmental pollution, as this will allow to understand the impact associated with industrial and anthropogenic activities as well as to make better decisions.

The aim of this study was to generate a spatial exposure model to identify risk zones in four pilot municipalities of the Mezquital Valley based on environmental variables such as environmental pollution factors and their relationship with the presence of certain diseases such as ischemic cardiomyopathy, mesothelioma, and pneumoconiosis, which may be caused by the main exposure routes (air and water).

## 2. Materials and Methods

### 2.1. Methodological Development

**Figure 1:**
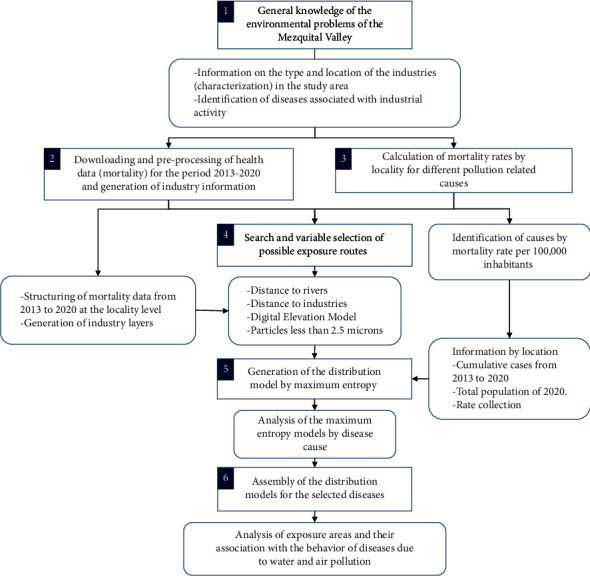
Methodological framework.

Figure 1 details the method used in the present study.

### 2.2. Study Area

**Figure 2:**
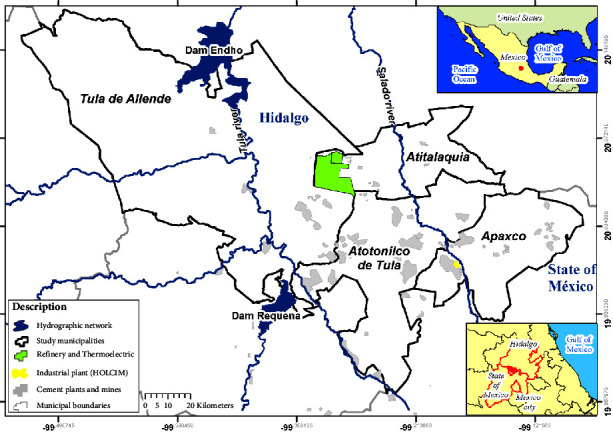
Study area.

The study area comprised four municipalities of the Health and Environmental Emergency Region (RESA in Spanish) situated within the State of Hidalgo and one in the State of Mexico (Figure 2). The population density concentrated across the four municipalities is shown in Table 1. The municipalities of Apaxco and Atotonilco share the same type of industry, i.e., mining. However, because Apaxco has a larger territorial extension, this was accounted for this municipality (Table 1).

The Mezquital Valley, over the years, has been considered as “an environmental hell” because in addition to the different industries, agricultural production, in the area, significantly contributes to development of diseases, such as cardiovascular, respiratory, and neoplastic diseases [25].

The contamination records of the study area are vast and date as far back as to 1881 with the installation of cement plants in 1890. In the 1970s, transport of wastewater from Mexico City began and the Miguel Hidalgo Refinery started operations whereas 1999–2003 saw the construction of the Francisco Pérez Thermoelectric Plant. Therefore, the mortality information for this study was obtained from the records provided by the Ministry of Health (https://www.dgis.salud.gob.mx/contenidos/basesdedatos/da_defunciones_gobmx.html). At the municipal level, the information was analyzed for the period 2000–2020 whereas at the local level, the period taken was from 2013 to 2020.

### 2.3. Rate Calculation for the Analysis of Diseases in the Mezquital Valley from 2000 to 2020

The rate calculation consists on dividing the number of events that occurred in a period and a population by the total person-time (the sum of the individual disease-free periods) in which the populations had the risk of presenting the event. The rate is expressed by multiplying the result per 100 000 population to allow comparisons [26].

**Figure 3:**
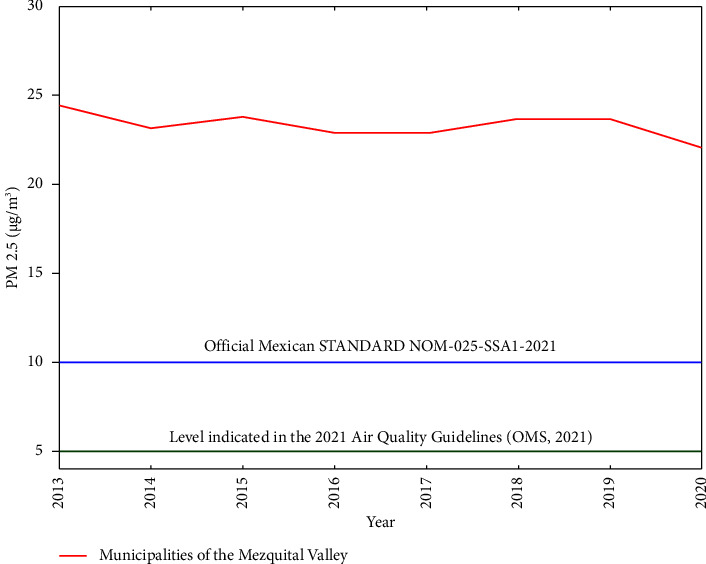
Annual pollution level by particulate matter (PM) 2.5.

A spatiotemporal analysis was carried out for all causes at the country level during a period from 2000 to 2020, with the aim of calculating the rates and making a comparison between what was obtained above the national value and the pilot municipalities that represented the study area. In addition, the frequency of each of the causes for each of the analyzed years was calculated allowing identifying both the main 20 causes presented during the study period and the diseases that are recurrent and associated with the contamination presented in the Mezquital Valley. According to the World Health Organization's global guidelines of the air quality, the 2.5 PM permissible limits are 5 *µ*g/m^3^ per year [27]; however, in Mexico, according to the NOM, they are 10 *µ*g/m^3^ per year [28]. When these limits are exceeded, they can cause damage to health. As seen in Figure 3, these values are above 22 *µ*g/m^3^ per year in the region of the Mezquital Valley.

This was done considering that the analyzed diseases, due to their behavior, can occur in the long term.

A comparative analysis was carried out between the mortality rate causes for the Mezquital Valley and the mortality rate causes at a national level through the calculation of the mortality rate ratio (MRR). This was done with the purpose of identifying those causes that often, during the period of analysis, present an MRR >1 which allowed the selection of determinant causes, such as ischemic cardiomyopathy (3.1), mesothelioma (2.3), and pneumoconiosis (3.7), as well as the frequency in years for 10, 11, and 14, respectively, as they are closely related to environmental pollution. Diabetes was excluded from the analysis because this disease is caused by different factors and not just by pollution.

### 2.4. Pollution Factors

**Figure 4:**
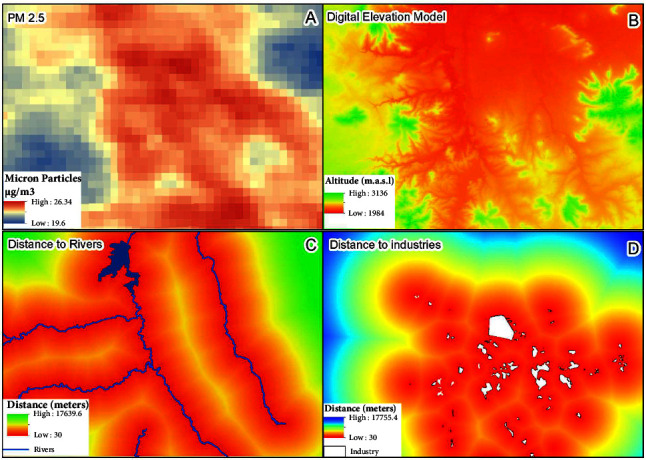
Pollution factors.

Particulate matter (PM 2.5) (Figure 4(a)) originates from directly emitted anthropogenic and natural sources and it is divided into fixed and mobile. These particles can contain pollutants such as SO_2_ and NO_2_ [29]. The information was downloaded from https://sites.wustl.edu/acag/. The product is an estimation of particulate matter, on an annual and monthly basis, at the ground level represented by surface images of approximately 1 km for the period 1998–2021.

The surface images are generated, through satellite remote sensing, from the understanding of the processes that affect tropospheric ozone and aerosols, combining the Aerosol Optical Depth (AOD) of the MODIS, MISR, and SeaWIFS instruments from the National Aeronautics and Space Administration (NASA) with the GEOS-Chem chemical transport model. The images are calibrated with global Earth observations using a geographically weighted regression (GWR) [30]. Two atmospheric monitoring stations with limited availability of information (<20%) were identified within the study area which did not allow validation of the product.

The digital elevation model (DEM) from the Shuttle Radar Topography Mission [31] with a resolution of 90 m (Figure 4(b)) was another variable to consider in this work. The variable for the rivers and bodies of water was obtained from CONABIO at https://www.conabio.gob.mx/informacion/gis/ in ^∗^.vct format. Buffers were generated from these because it was important to have the distance to rivers as sources of pollution as the populations closest to these rivers are those with the highest exposure (Figure 4(c)).

In order to obtain the geospatial information about the industries that exist in the Mezquital Valley, a literature review was done to generate a list of the industries that exist here. At the same time, a processing process was carried out using Sentinel satellite images (https://scihub.copernicus.eu/) to have the most up-to-date geospatial information (^∗^.vct) about all industries that generate some type of environmental impact, such as refineries, thermoelectric and cement plants, and microindustries related to the textile industry. In this layer, it was also relevant to obtain the distance through a continuous image by drawing distance buffers, with the aim of showing that the communities closest to these fixed pollution sources are those with the greatest exposure (Figure 4(d)).

### 2.5. Generation of the Maximum Entropy Model

The maximum entropy model with the MaxEnt algorithm version 3.3.3k [32] was used for regionalization of the population by areas of exposure to pollutants. Mortality causes by location associated with the 4 previously identified contamination factors were used as occurrence data. Their independence was evaluated with the Pearson correlation coefficient (*r* < 0.85).

### 2.6. Classification for Jenks Natural Breaks Algorithm

The Natural Breaks (Jenks natural breaks algorithm) classification method within the ArcGis 10.5 Reclassify Spatial tool was used to calculate the probability model for exposure to pollutants with health risks. This method performs data clustering by minimizing the within-group variance and maximizing the between-group variance [33, 34]. This was regionalized into the following three frequency distribution categories: low exposure: 0.01–0.38, medium exposure: 0.38–0.55, and very high exposure: 0.55–1.

### 2.7. Odds Ratio for the Association of the Geographic Distribution Model of Cases and Incidence of Diseases

Odds ratios were calculated to evaluate the disease distribution of cases within the exposure categories. This association type has been used in different studies to measure the relationship between two events that occurred in a population group [35]. This process was carried out using the IBM SPSS statistical software.

The total number of cases and population by exposure region was obtained to calculate the odds ratio. The equation applied was OR = (*A*: cases exposed × *D*: noncases exposed)/(*B*: cases unexposed × *C*: noncases unexposed), where very high exposure is the number of exposed cases and both medium and low exposure represent the number of nonexposed cases. A value <1 would indicate a protective behavior, that is, the pollution factors chosen protect against the occurrence of mortality cases. On the contrary, a value >1 would indicate an association between the exposure categories and the pollution factors [36].

### 2.8. Attributable Risk by Cause

Relative risk is a ratio of two probabilities between the likelihood of having the disease in the exposed population over the probability of not having it in the unexposed one; hence, it is expressed as the comparison of the incidence rate of the disease between the exposed and nonexposed municipalities [37].

## 3. Results and Discussion

### 3.1. Results

The behavior of some of the causes was analyzed on an annual basis (2000–2020), as an exploratory result, identifying the presence of 300–500 deaths per year. At the same time, the top 20 pollution-related diseases for the study area were identified. Ischemic cardiomyopathy had a frequency of 10 years and a MRR three times higher than the national rate, mesothelioma presented a frequency of 11 years with an MRR = 2.3, whilst pneumoconiosis' frequency was 14 years with an MRR = 3.7 (Table 2).

This first stage resulted in the generation of a more detailed analysis of specific causes related to both water and air pollution sources, which allowed the creation of a locality-level analysis. As mentioned above, the analyzed period is narrowed to 2013–2020 due to data availability. Some variants were considered per each cause that according to their behavior can be related to the pollution generated by current industries in the Mezquital Valley. The chosen causes are shown in Table 2, which reveals that the condition with the highest rate value is ischemic cardiomyopathy with 38.9 per 100,000 inhabitants, followed by mesothelioma with 2.5 and pneumoconiosis with 0.96. It is worth mentioning that the 2020 population census was used in order to consider the entire population exposed in the 2013–2020 period with mortality information by locality.

**Figure 5:**
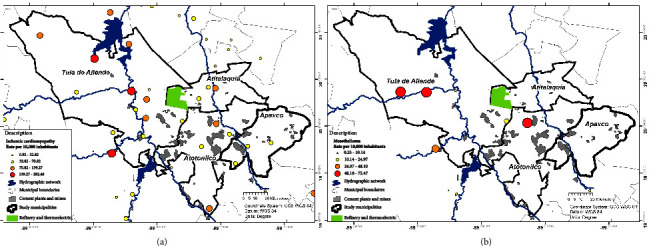
Rates for ischemic cardiomyopathy (a) and mesothelioma (b).

Presence data were obtained from the cases distributed by locality. A higher number of cases per locality was seen in the case of ischemic cardiomyopathy (Figure 5(a)) and, therefore, a greater distribution, with rate values ranging from 132 to 289 cases per 100,000 inhabitants in the western part of the study area which correlates to the course of the rivers that pass-through the Tula de Allende locality and some other localities near the cement plants. On the other hand, mesothelioma (Figure 5(b)) was found in fewer localities with its highest values between 48 and 75 cases per 100,000 inhabitants; however, two clusters were identified one near the river border in Tula de Allende and the second nearby, between the refinery and cement plants in the Atitalaquia and Atotonilco de Tula municipalities.

The spatial distribution of mortality rates by locality mainly occurs on proximity to rivers and dams. This was the variable that most influenced the model, followed by the Shuttle Radar Topography Mission (SRTM), which marks the area's topographic characteristics and influences the winds' direction and, therefore, affects the dispersion of pollution in both air and water (Figure 5).

### 3.2. Analysis of the Ischemic Cardiomyopathy Model

The model showed a AUC value of 0.82 and 0.85 for ischemic cardiomyopathy and mesothelioma, respectively, thus indicating that the prediction has good reliability (AUC <1). The disease suitability is associated with pollution factors [38].

The variables with the highest percentage of contribution to this ischemic cardiomyopathy model were the rivers with 71.3%, followed by the digital elevation model with 13.6%, therefore indicating the area's orographic characteristics can influence in the wind direction. The distance to industries contributed 8.2%.

**Figure 6:**
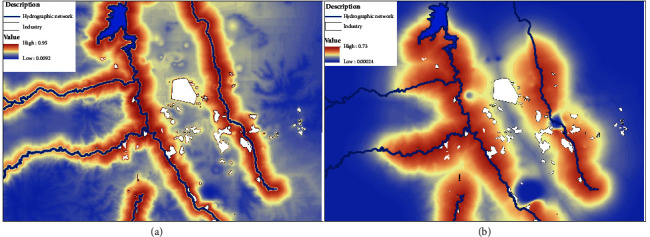
Distribution model for ischemic cardiomyopathy (a) and mesothelioma (b).

According to the model's results, the area with the highest probability of ischemic cardiomyopathy occurrence is very close to the rivers that have been contaminated by wastewater coming from Mexico City's metropolitan area which crosses the industrial zone of the Mezquital Valley in conjunction with the increase in the distribution of atmospheric pollution particles above 20 *µ*g/m^3^. This pollution can take time to dissipate due to the area's orography as it is located in areas of low altitudes; therefore, the convergence of these factors (Figure 4) generates an impact on the population (Figure 6(a)).

### 3.3. Analysis of the Mesothelioma Model

The mesothelioma model showed a similar behavior to that of the ischemic cardiomyopathy, but with greater restriction. Cases were located in close proximity to rivers and to the most polluting industries and cement plants. The latter are considered to have the highest exposure (Figure 6(b)).

The variables involved in the mesothelioma model showed that the distance to rivers contributed 63.5%, whereas the distance to industries contributed 30.1%. This may be due to the fact that the disease is caused by direct exposure (inhalation or ingestion) to asbestos or by living with individuals who work with this mineral, which, in many cases, is associated with industrial activity.

### 3.4. Spatial Distribution of Disease Risk

The analysis of the areas of the two generated models allowed to detailed the data distribution of the disease's presence in relation to the proximity to pollution factors such as rivers and bodies of water, which are, on average, about 1700 meters the above sea level and present high concentrations of PM 2.5 with average values above 25 *µ*g/m^3^. According to the Official Journal of the Federation 2021 (DOF in Spanish) [28] and the NOM-025-SSA1-2021 for Environmental Health values above 10 *µ*g/m^3^ can result in diseases of the central nervous system, metabolic syndrome, and kidney dysfunctions.

Moreover, the ATSDR [39] reports that an average annual exposure greater than 24 *µ*g/m^3^ is associated, in the short term, to mortality from ischemic cardiomyopathy and, in the long term, with different types of cancer.

**Figure 7:**
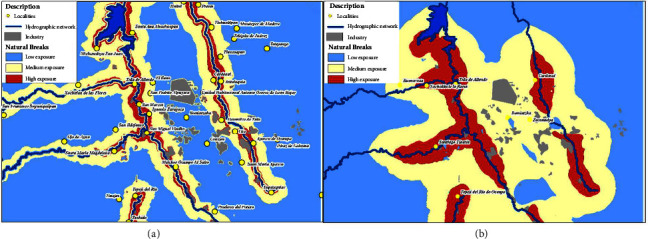
Model of exposure zones for ischemic cardiomyopathy (a) and mesothelioma (b).

The cases for ischemic cardiomyopathy and mesothelioma were dispersedly concentrated within the study area with 201 mortality records for ischemic cardiomyopathy and 13 for mesothelioma (Figure 7(a)). The cases distribution and rate values per 100,000 inhabitants for ischemic cardiomyopathy matched the different exposure areas. The highest values were obtained in the high exposure area as seen in Table 3; therefore, this shows that there is an association with the pollution factors present in the study area. However, this is not the case for the low exposure category as the relationship with the proximity to industries is considered unlikely although the north to southeast wind direction could contribute to an increase in air pollution which affects from the center part outwards of the Mezquital Valley [29, 40].

The category model of mesothelioma disease (Figure 7(b)) showed cases in close proximity to rivers and in the central part which is where the industries are. However, in the very high exposure zone, a rate value of 4.72 cases per 100,000 inhabitants was identified. This is the most significant value compared to both the medium exposure (1.04 rate value) and low exposure (0.88 rate value) categories (Table 3).

## 4. Discussion

**Figure 8:**
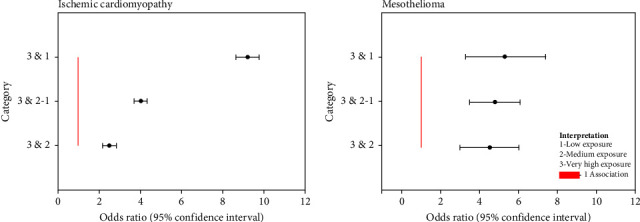
Odds ratios by exposure category with confidence interval for ischemic cardiomyopathy and mesothelioma.

The number of cases and association measures for ischemic cardiomyopathy are observed by exposure area (Table 3). The high exposure area showed that the rate value was the highest, which reflects an association level of the disease presence with the pollution factors in comparison to the medium and low exposure zones which value was >1 (OR = 4.03 and 95% CI: 3.72–4.33). When the association is compared with the low exposure zone, the association value increases (OR = 9.22 and 95% CI: 8.65–9.79) (Figure 8(a)).

In relation to the association measures for mesothelioma, its odds ratio value was also >1 (Table 4) because when compared, the very high exposure zone versus the medium and low exposure zone, OR = 4.79 (95% CI: 3.50–6.08) whereas between the high exposure zone and the low exposure zone, OR = 5.35 (95% CI: 3.29–7.40) (Figure 8(b)).

The results shown in this study demonstrate a relationship between environmental pollution and the analyzed diseases. Nawrot et al. [41] supported this with their findings that mention that one of the triggering factors of heart attacks is air pollution. The same was observed for the mesothelioma disease, where Carbone et al. [42] stated that this disease is related to occupational exposure to asbestos or asbestos carcinogenic fibers present in the environment in developing countries despite its prohibition since the 70s. However, nowadays, asbestos are still used in the industrial sector, such as in cement plants affecting workers and people due to exposure from washing clothes or cleaning objects contaminated with them [43, 44].

### 4.1. Attributable Risk

**Figure 9:**
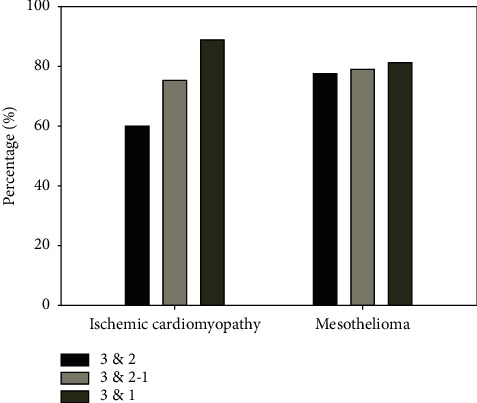
Attributable risk. 1: low exposure, 2: medium exposure, 3: very high exposure.

The attributable risk result for ischemic cardiomyopathy and mesothelioma (Figure 9) between the high exposure zone versus the medium and lower exposure zones showed how much these diseases are attributable to activities related to pollution sources in rivers, industrial activities, or even within the study area that presents these types of problems that affect the population.

Both ischemic cardiomyopathy and mesothelioma are attributable to pollution. The zone that presented the highest risk for ischemic cardiomyopathy and mesothelioma was the very high exposure zone versus low exposure zone with values of 89% and 81%, respectively, whereas the very high exposure zone versus the medium and low exposure zone had 75% and 79% for each disease. Finally, the very high exposure zone versus medium exposure zone presented 59% for ischemic cardiomyopathy and 77% for mesothelioma. These results show that if pollution is reduced or if policies to reduce pollution are implemented the appearance of these two diseases could be prevented.

Contreras et al. [45] mentioned that there is no direct exposure due solely to agricultural activities, rather indirect exposure routes need to be identified, since these are responsible for the increased risk of disease. The author also proposes that these exposure routes could include consumption of crops irrigated by wastewater, contact with domestic animals, flooding events, and the spread of fecal matter by pathogens. This agrees with the statement supported by various studies which mention that pollution can occur through different exposure pathways, such as water and air [9, 46].

Several institutions are currently carrying out important prevention initiatives related to pollution and the development of cardiovascular diseases among which the Spanish Society of Cardiology and the Spanish Heart Foundation with the SEC-FEC project stand out. Their work is crucial for understanding of environmental pollution as a risk factor and as an initiative to address a public health challenge [47].

Currently, environmental pollution has not been addressed by government agencies and collegiate groups (NOMs and disease control). Prolonged exposure to environmental pollution can generate various diseases. The implementation of strategies to reduce toxicity, like the application of chelation therapy that eliminates the presence of heavy metals in the bloodstream [48] could be an alternative. At the same time, the reduction and monitoring of environmental pollution could help to enhance prevention and to reduce the number of deaths from diseases such as cardiovascular, mesothelioma, and pneumoconiosis in addition to reducing other impacts such as those of climate change [49].

Ischemic cardiomyopathy is a disease that can be related to quality of life (diet, lack of exercise, and obesity) and mobile sources [50], whereas mesothelioma can be related to radiotherapy (chest cancer) and exposure to asbestos. Pneumoconiosis, on the other hand, is related to tobacco consumption and exposure to environments with excessive concentrations of inorganic particles. This study has exposed a diversity of epidemiological studies that show a strong association between pollution and the diseases presented; however, the official information collected from the health sector does not specify the clinical characteristics by which these types of diseases are acquired, so it is complicated to distinguish the confounding factors, and more detailed studies are required to rule out these factors [51].

## 5. Conclusions

Ischemic cardiomyopathy and mesothelioma showed a distribution associated with the main pollution sources, such as polluted rivers and bodies of water, and industries that act as a pollution source. Environmental pollution is a risk factor for ischemic cardiomyopathy, as small pollution particles enter the bloodstream and damage the blood vessels' inner walls which in turn causes their reduction and hardening as well as blood pressure rise. This could lead to formation of clots and ultimately death due to a heart attack or stroke.

The environmental variables that contributed to the generation of the ischemic cardiomyopathy model showed that the combination between distance to rivers, orography, distance to industries, and the PM 2.5 collectively have an influence in the presence of this disease. This is mainly due to the pollution that is concentrated within the study area because low wind speed and the orographic barriers cause poor dispersion of pollutants.

Mesothelioma is a disease caused by either ingestion and/or inhalation of asbestos or by living with people who work with this mineral. This type of activity is directly related to the cement industry and the manufacturing of paper products and some automotive components. There are different cement factories located in the Mezquital Valley area, including some of the most important in the country.

Pneumoconiosis was the disease that presented less cases; however, these cases occurred in the zone of greatest exposure; therefore, the results between the medium and low exposure zones were not comparable. Finally, among the analyzed causes, mesothelioma showed the highest association to the exposure zones based on its odds ratio value.

Modeling of the cases distribution, based on the results from this study, is a useful tool that can help public health authorities, government agencies, and communities to identify and to monitor diseases related to areas at high risk of pollution. The availability of information in the consulted databases allowed correlating pollution factors with the confirmed cases for mesothelioma, ischemic cardiomyopathy, and pneumoconiosis. Therefore, the model based on maximum entropy was suitable for the identification of pollution-affected areas, as well as the main pollution sources that led to the presence of these diseases.

As a result of this investigation, an update about the information on the industries, cases, and their association to pollution factors based on a detailed model that specifies the locations with the greatest exposure was generated. The findings from this study, particularly those regarding the behavior among PM2.5, could serve as a tool to create regulatory proposals and to reduce the emissions from pollution sources.

## Figures and Tables

**Table 1:** Population in 2020 by municipality in the Mezquital Valley [24].

Municipalities	Population	Population density (per km^2^)	Number of industries
Atitalaquia	31,525	508.54	4
Atotonilco de Tula	62,470	514.36	14
Tula de Allende	115,107	344.97	8
Apaxco	31,898	424.62	12
Total	241,000		

**Table 2:** Total cases and rate per cause of death in the 4 municipalities of the RESA region of the Mezquital Valley (2013–2020).

Cause	Code CIE 10 variants	Localities	Total cases	Population 2020	Rate per 100,000
Ischemic cardiomyopathy	I250, I251, I252, I253, I254, I255, I256, I258, I259	42	201	515, 726	38.9
Mesothelioma	C450, C451, C452, C457, C459	9	13	515, 726	2.5
Pneumoconiosis	J60, J61, J62, J63, J620, J628, J632, J633, J634, J635, J638, J64x	3	5	515, 726	0.96

**Table 3:** Zones by exposure area for ischemic cardiomyopathy and mesothelioma.

Exposure zone	Ischemic cardiomyopathy			Mesothelioma		
	Rate x 100,000	Area (km^2^)	Number of localities	Rate x 100,000	Area (km^2^)	Number of localities
Low exposure	7.85	1159.7	9	0.88	864.62	1
Medium exposure	28.99	381.57	21	1.04	548.4	2
High exposure	72.43	64.68	12	4.72	193	6

**Table 4:** Cases association by the exposure zone for ischemic cardiomyopathy and mesothelioma.

Exposure zone	Ischemic cardiomyopathy			Mesothelioma		
	Odds ratio	Confidence interval (95%)		Odds ratio	Confidence interval (95%)	
		IC−	IC+		IC−	IC+
High exposure vs. low exposure	9.22	8.65	9.79	5.35	3.29	7.40
High exposure vs. medium and low exposure	4.03	3.72	4.33	4.79	3.50	6.08
High exposure vs. medium exposure	2.49	2.16	2.83	4.52	3.0	6.04

## Data Availability

The data that support the findings in this study are available from the corresponding author upon request.
